# Commentary: Aberrant dynamic functional connectivity of posterior cingulate cortex subregions in major depressive disorder with suicidal ideation

**DOI:** 10.3389/fnins.2022.1012050

**Published:** 2022-09-16

**Authors:** Zongling He, Fengmei Lu

**Affiliations:** ^1^The Fourth People's Hospital of Chengdu, Chengdu, China; ^2^Chengdu Mental Health Center, Chengdu, China

**Keywords:** dynamic functional connectivity (dFC), major depressive disorder (MDD), suicidal ideation (SI), posterior cingulate cortex (PCC), resting-state functional magnetic resonance imaging (fMRI)

Major depressive disorder (MDD) is a well-known mental disorder which influences millions of individuals worldwide (Ferrari et al., 2013). MDD not only leads to severe mental and physical disabilities but also cause a high risk for suicide (Wang et al., 2017; Dong et al., 2018). It is reported that 58% of MDD patients carry suicidal ideation (SI) and 15% of them have attempted suicide (Sokero et al., 2003). More than 50% of Chinese MDD patients have SI (Fang et al., 2018). Thus, to better understand the neural mechanisms underlying SI in MDD is of necessary and urgent significance for the progress in suicide prevention and intervention.

Recently, evidence demonstrates that changes in a wide range of brain regions and functional connectivity (FC) are associated with SI, such as the amygdala, the insula, the ventral and dorsal prefrontal cortex, the posterior cingulate cortex (PCC), the anterior cingulate cortex, the cortico-limbic-striatal FC, the orbitofrontal-thalamic FC, and the frontal-limbic FC (Du et al., 2017; Kim et al., 2017; Wei et al., 2018; Schmaal et al., 2020; Li et al., 2022b; Yang et al., 2022). Despite recent neuroimaging developments, FC abnormalities in MDD with SI are still poorly understood. Moreover, most FC studies exploring static FC on MDD with SI relied on the implicit assumption that the degree of connectivity strength among regions remains stationary over time. However, studies have indicated that our brain is not static, but rather is inherently dynamic (Allen et al., 2014; Lu et al., 2020, 2021). Till now, few studies examined the dynamic FC in MDD with SI. For example, Liao et al. showed that MDD with SI revealed increased dynamic connectomics compared with MDD without SI and healthy controls (HCs) (Liao et al., 2018). Qiao et al. reported changed dFC between habenula and other brain regions in MDD with SI relative to MDD without SI and HCs (Qiao et al., 2020). Those studies indicated that altered dFC could help distinguish patients with SI from those without SI as compared with static FC. Hence, to understand how FC between different brain regions strengthen or weaken over time could help provide novel insight into the neural communication in MDD with SI from the view of temporal stability.

The PCC was reported to play a vital role in the neural mechanisms in MDD patients with SI. Previous studies showed abnormal structural and functional activities in PCC as well as aberrant FC between PCC and other brain regions in MDD with SI (Marchand et al., 2013; Ambrosi et al., 2019; Schmaal et al., 2020; Hong et al., 2021). Moreover, Chase et al. demonstrated disrupted static FC in PCC subregions in MDD with SI (Chase et al., 2017, 2021). However, there are no studies investigating the dFC variability of the PCC subregions in MDD with SI.

Therefore, a recent study of Li et al. (2022a) is very timely. In their study, the authors supposed that (1) MDD with SI would show aberrant dFC variability in PCC subregions as compared with MDD without SI and HCs; and (2) the anomalous dFC variability would exhibit associations with clinical variables. To test their hypotheses, resting-state functional magnetic resonance imaging (rsfMRI) data were obtained from 31 unmedicated MDD with SI (SI), 56 unmedicated MDD without SI (NSI), and 48 matched HCs. The PCC was segmented into bilateral dorsal PCC (dPCC) and bilateral ventral PCC (vPCC) according to the Human Brainnetome Atlas. Then, the whole-brain dFC analysis of each PCC subregion was performed by using a sliding-window method with a window length of 50 TR (100 s) and a step size of 1 TR (2 s), resulting in 181 consecutive windows. After that, the dFC was measured by computing the standard deviation (SD) of the *z* maps across the 181 windows. Subsequently, analysis of covariance (ANCOVA) was applied to examine the between-group differences in dFC maps among the three groups for each PCC subregion with age, gender, and mean FD as covariates. Next, the mean *z*-scores of brain regions showing significant between-group differences were extracted for *post hoc* analyses. Finally, the Spearman correlation analysis was conducted between clinical variables and the abnormal dFC variability in MDD patients. The ANCOVA results showed altered dFC variability between the left dPCC and left fusiform gyrus, left vPCC and left inferior frontal gyrus (IFG), and the right vPCC and left IFG among the three groups. *Post hoc* analysis results demonstrated that SI group showed higher dFC variability between the left vPCC and left IFG in relative to NSI group, whereas the SI and NSI groups indicated increased dFC variability between the left dPCC and left fusiform gyrus, and right vPCC and left IFG than HCs. Further, the dFC variability between the left vPCC and left IFG was positively associated with the suicide ideation scores across all the MDD patients.

Overall, the study was the first to report aberrant dFC variability within PCC subregions in MDD with SI. Their findings revealed that the key brain regions involved in the cognition, negative self-perceptions, and negative emotion generation, as well as the default mode network and frontoparietal network were disrupted in MDD with SI, which contributes to advance our understanding of the potential neural mechanisms in MDD with SI from the perspective of dynamic FC. However, here, we want to point out several shortcomings about methodological issues. First, the educational level information was missed for the HCs. In the ANCOVA and *post hoc* analyses, the covariates should also include the educational level as well as the variance of FD. Second, we did not see the handedness information for participants. Thirdly, since SD will over-represent data used in the center of the run and are often dependent on the mean connectivity, we suggested that the authors could also compute the coefficient of variation (CV = SD/mean) to validate their results. Finally, in the correlation analysis, it is better to regress out the age, gender, mean FD, variance of FD, as well as the clinical information such as duration of illness, and age of onset. Another concern is that in the Figure 4, the results may be influenced by some outlier data points. In conclusion, future studies could include large sample size, more detailed demographic and clinical information, as well as more rigorous method to corroborate their findings.

## Author contributions

ZH and FL conceived the idea and wrote the manuscript. All authors contributed to the article and approved the submitted version.

## Funding

This study was supported by the National Natural Science Foundation of China (62006038, 62173069, and 82101620) and Sichuan Province Science and Technology Support Program (2022YFS0180 and 2022NSFSC0590).

## Conflict of interest

The authors declare that the research was conducted in the absence of any commercial or financial relationships that could be construed as a potential conflict of interest.

## Publisher's note

All claims expressed in this article are solely those of the authors and do not necessarily represent those of their affiliated organizations, or those of the publisher, the editors and the reviewers. Any product that may be evaluated in this article, or claim that may be made by its manufacturer, is not guaranteed or endorsed by the publisher.

## References

[B1] Allen E. A. Damaraju E. Plis S. M. Erhardt E. B. Eichele T. Calhoun V. D. (2014). Tracking whole-brain connectivity dynamics in the resting state. Cerebral cortex 24, 663–676. 10.1093/cercor/bhs352 23146964 PMC3920766

[B2] Ambrosi E. Arciniegas D. B. Curtis K. N. Patriquin M. A. Spalletta G. Sani G.. (2019). Resting-state functional connectivity of the habenula in mood disorder patients with and without suicide-related behaviors. J. Neuropsy. Clin. neurosciences 31, 49–56. 10.1176/appi.neuropsych.17120351 30282513 PMC6697145

[B3] Chase H. W. Auerbach R. P. Brent D. A. Posner J. Weissman M. M. Talati A. (2021). Dissociating default mode network resting state markers of suicide from familial risk factors for depression. Neuropsychopharmacology 46, 1830–1838. 10.1038/s41386-021-01022-5 34059799 PMC8358011

[B4] Chase H. W. Segreti A. M. Keller T. A. Cherkassky V. L. Just M. A. Pan L. A.. (2017). Alterations of functional connectivity and intrinsic activity within the cingulate cortex of suicidal ideators. J. Affect. Diso. 212, 78–85. 10.1016/j.jad.2017.01.013 28157550 PMC5358995

[B5] Dong M. Wang S.-B. Li Y. Xu D.-D. Ungvari G. S. Ng C. H.. (2018). Prevalence of suicidal behaviors in patients with major depressive disorder in China: a comprehensive meta-analysis. J. Affect. Disord. 225, 32–39. 10.1016/j.jad.2017.07.043 28779680

[B6] Du L. Zeng J. Liu H. Tang D. Meng H. Li Y.. (2017). Fronto-limbic disconnection in depressed patients with suicidal ideation: A resting-state functional connectivity study. J Affect Disord 215, 213–217. 10.1016/j.jad.2017.02.027 28340447

[B7] Fang X. Zhang C. Wu Z. Peng D. Xia W. Xu J.. (2018). Prevalence, risk factors and clinical characteristics of suicidal ideation in Chinese patients with depression. J Affect. Disord. 235, 135–141. 10.1016/j.jad.2018.04.027 29655075

[B8] Ferrari A. J. Charlson F. J. Norman R. E. Patten S. B. Freedman G. Murray C. J.. (2013). Burden of depressive disorders by country, sex, age, and year: findings from the global burden of disease study 2010. PLoS Med. 10, e1001547. 10.1371/journal.pmed.1001547 24223526 PMC3818162

[B9] Hong S. Liu Y. S. Cao B. Cao J. Ai M. Chen J.. (2021). Identification of suicidality in adolescent major depressive disorder patients using sMRI: A machine learning approach. J. Affect. Disord. 280, 72–76. 10.1016/j.jad.2020.10.077 33202340

[B10] Kim K. Kim S.-W. Myung W. Han C. E. Fava M. Mischoulon D.. (2017). Reduced orbitofrontal-thalamic functional connectivity related to suicidal ideation in patients with major depressive disorder. Sci. Rep. 7, 1–11. 10.1038/s41598-017-15926-0 29150619 PMC5693996

[B11] Li W. Wang C. Lan X. Fu L. Zhang F. Ye Y.. (2022a). Aberrant dynamic functional connectivity of posterior cingulate cortex subregions in major depressive disorder with suicidal ideation. Front. Neurosci. 16, 937145. 10.3389/fnins.2022.937145 35928017 PMC9344055

[B12] Li W. Wang C. Lan X. Fu L. Zhang F. Ye Y.. (2022b). Resting-state functional connectivity of the amygdala in major depressive disorder with suicidal ideation. J. Psych. Res. 153, 189–196. 10.1016/j.jpsychires.2022.07.001 35839660

[B13] Liao W. Li J. Duan X. Cui Q. Chen H. Chen H. (2018). Static and dynamic connectomics differentiate between depressed patients with and without suicidal ideation. Hum. Brain Mapp. 39, 4105–4118. 10.1002/hbm.24235 29962025 PMC6866497

[B14] Lu F. Liu P. Chen H. Wang M. Xu S. Yuan Z.. (2020). More than just statics: Abnormal dynamic amplitude of low-frequency fluctuation in adolescent patients with pure conduct disorder. J. Psychiatr. Res. 131, 60–68. 10.1016/j.jpsychires.2020.08.027 32937251

[B15] Lu F. Zhao Y. He Z. Ma X. Yao X. Liu P.. (2021). Altered dynamic regional homogeneity in patients with conduct disorder. Neuropsychologia 157, 107865. 10.1016/j.neuropsychologia.2021.107865 33894243

[B16] Marchand W. R. Lee J. N. Johnson S. Gale P. Thatcher J. (2013). Differences in functional connectivity in major depression versus bipolar II depression. J. Affect. Disord. 150, 527–532. 10.1016/j.jad.2013.01.028 23433856

[B17] Qiao D. Zhang A. Sun N. Yang C. Li J. Zhao T.. (2020). Altered static and dynamic functional connectivity of habenula associated with suicidal ideation in first-episode, drug-naïve patients with major depressive disorder. Front. Psych. 11, 608197. 10.3389/fpsyt.2020.608197 33391057 PMC7772142

[B18] Schmaal L. van Harmelen A.-L. Chatzi V. Lippard E. T. Toenders Y. J. Averill L. A.. (2020). Imaging suicidal thoughts and behaviors: a comprehensive review of 2 decades of neuroimaging studies. Molec. Psychiat. 25, 408–427. 10.1038/s41380-019-0587-x 31787757 PMC6974434

[B19] Sokero T. P. Melartin T. K. Rytsälä H. J. Leskelä U. S. Lestelä-Mielonen P. S. Isometsä E. T. (2003). Suicidal ideation and attempts among psychiatric patients with major depressive disorder. J. Clin. Psychiatry 64, 1094–1100. 10.4088/JCP.v64n0916 14628986

[B20] Wang J. Wu X. Lai W. Long E. Zhang X. Li W.. (2017). Prevalence of depression and depressive symptoms among outpatients: a systematic review and meta-analysis. BMJ Open 7, e017173. 10.1136/bmjopen-2017-017173 28838903 PMC5640125

[B21] Wei S. Chang M. Zhang R. Jiang X. Wang F. Tang Y. (2018). Amygdala functional connectivity in female patients with major depressive disorder with and without suicidal ideation. Ann. General Psychiat. 17, 1–7. 10.1186/s12991-018-0208-0 30214465 PMC6134510

[B22] Yang J. Palaniyappan L. Xi C. Cheng Y. Fan Z. Chen C.. (2022). Aberrant integrity of the cortico-limbic-striatal circuit in major depressive disorder with suicidal ideation. J. Psychiatr. Res. 148, 277–285. 10.1016/j.jpsychires.2022.02.003 35180634

